# Component-Wise Error Correction Method for UWB-Based Localization in Target-Following Mobile Robot

**DOI:** 10.3390/s22031180

**Published:** 2022-02-04

**Authors:** Kyungbin Bae, Yooha Son, Young-Eun Song, Hoeryong Jung

**Affiliations:** 1 Division of Mechanical and Aerospace Engineering, Konkuk University, 120 Neungdong-ro, Gwangjin-gu, Seoul 05029, Korea; bkb126@konkuk.ac.kr (K.B.); ssroom9@konkuk.ac.kr (Y.S.); 2 Department of Electrical Engineering, Hoseo University, 20 Hoseo-ro 79 beon-gil, Baebang-eup, Asan-si 31499, Korea; tdsong@hoseo.edu

**Keywords:** autonomous mobile robot, UWB localization, target tracking

## Abstract

Target-following mobile robots have gained attention in various industrial applications. This study proposes an ultra-wideband-based target localization method that provides highly accurate and robust target tracking performance for a following robot. Based on the least square approximation framework, the proposed method improves localization accuracy by compensating localization bias and high-frequency deviations component by component. Initial calibration method is proposed to measure the device-dependent localization bias, which enables a compensation of the bias error not only at the calibration points, but also at the any other points. An iterative complementary filter, which recursively produces optimal estimation for each timeframe as a weighted sum of previous and current estimation depending on the reliability of each estimation, is proposed to reduce the deviation of the localization error. The performance of the proposed method is validated using simulations and experiments. Both the magnitude and deviation of the localization error were significantly improved by up to 77 and 51%, respectively, compared with the previous method.

## 1. Introduction

Recently, human-following mobile robots have been introduced to ease the burden of human operators in various applications [1,2,3]. Robust and reliable human tracking is a key technology of following robots, and it enables mobile robots to operate in cooperation with humans. Following robots have been successfully applied in several domains, including logistics [4,5], shopping [6,7,8], and smart factories [9,10,11]; however, they are not technically mature and require further technical development for robust and reliable human tracking.

Human-tracking methods can be classified depending on the type of sensors used to measure the location of a target person. Camera vision was generally adopted in previous studies because it provides abundant scene information with relatively low cost [12]. However, the absence of depth information, a narrow field of view, and malfunctions due to illumination changes make it difficult to use only camera vision for tracking. Depth cameras and LiDAR have been used to supplement camera vision to provide three-dimensional point clouds of a target object. Previous studies have proposed human-tracking methods that combine camera vision with depth camera or LiDAR data [13,14]. However, these sensor-fusion methods also have a critical limitation because tracking failure can frequently occur in crowed environments when the camera loses the target when it is hidden by opaque obstacles.

A wireless-communication-based indoor positioning system (IPS) can be used to robustly measure the position of the target person regardless of surrounding obstacles. Even though the accuracy may be affected by the obstacles, the system does not lose the target. Wireless LAN (WLAN) [15], Bluetooth [16], and Zigbee [17] have been used for indoor positioning in various applications, but meter-level positioning errors (>1 m) prevent the use of these systems for human tracking in following robots. Ultra-wideband (UWB)-based IPS, which uses frequency bands ranging from 3.1 to 10.6 GHz with a broad occupancy bandwidth of more than 20% of the central frequency, provides a more accurate position measurement (<30 cm) compared with other IPS methods, and it can be adopted in the following robot as an effective sensor system for human tracking.

UWB-based IPS is composed of multiple UWB transceiver modules that are stationarily installed in the environment, called anchors, and a transceiver module installed on a moving target, called a tag. The system estimates the position of the tag using the given distances between the tag and each anchor, which are calculated using the time-of-flight of UWB communication. Previous studies describe two types of approaches to reduce positioning errors under the given errors in a distance measurement, i.e., fingerprinting and geometric approaches. In the fingerprinting approaches, machine learning techniques are used to estimate the position of the tag using empirical datasets, which consist of experimentally measured UWB transceiver signals and corresponding true locations [18,19]. It provides accurate position estimation for pre-trained environments, but it is difficult to use in practical applications because the positioning accuracies are too dependent on the training datasets, which are not applicable to unknown environments. The geometric approaches estimate the position of the tag utilizing the geometrical relations between the transceivers. The geometric approaches estimate the position of the tag utilizing the geometrical relations between the transceivers. In the literature, several methods have been proposed including receive signal strength (RSS), angle-of-arrival (AOA), time-difference of arrival (TDOA), and time-of-arrival (TOA) [20]. The geometric approaches can be classified into two categories, i.e., parametric and nonparametric methods, according to the description of the error characteristics in the distance measurement [18]. The parametric method defines the error characteristics using a probability density function, and it requires a sufficiently large number of samples to ensure the reliability of the probability density function. Maximum likelihood [21,22] and Bayesian estimation [23] have been proposed for the precise parameterization of the error that results in an improvement of the positioning accuracy; however, the accuracy can be critically degraded by variations in the error characteristics. The nonparametric method, which determines the error characteristics based on descriptive statistics, provides a position estimation that is robust to the uncertainties in the errors. Even though the parametric method may demonstrate superior performance in specific cases, the nonparametric method is more appropriate for the UWB-based IPS, owing to device-dependent errors in the UWB-based distance measurement, such as clock drift, frequency drift, and a timestamp that is difficult to represent as a probability density function [24].

A typical example of the nonparametric method is the least square (LS) approximation. In a practical situation, the LS approximation sensitively responds to non-line-of-sight (NLOS) situations and device-dependent measurement errors [25]. The weighted least square (WLS) approximation, which introduces weight factors to the LS, was proposed to improve the performance of the LS approximation [26,27]; however, it experiences difficulties in determining the weight factors based on the covariance of errors. Residual weighted least square (RWLS) approximation, which evaluates the reliability of data using residuals rather than weight factors, has been proposed to address this problem [27]. The LS-based approximations find the optimal position estimation inside a particular area bound that covers possible distance-measurement errors. Unlike the LS-based approximation, the modified hyperbolic (M-HB) algorithm estimates the position of the tag by investigating possible intersection points between lines that connect the points of the tag to each anchor [28]. It uses distance data from two of the three anchors to create two intersections that are probable tag positions, and the remaining anchor determines the correct intersection between the two. However, M-HB has difficulty in identifying the exact position of the tag in the case where the distances between the remaining anchor and the two intersections are same, thereby limiting its angle of use.

Previous UWB-based positioning methods assumed that the UWB transceivers are configured as a UWB tag moves in the area enclosed by multiple (>3) UWB anchors that are stationarily installed in the environment. However, the configuration of the UWB transceivers should be modified for human tracking in the following robot. The UWB anchors should be mounted on the robot because it aims to identify the position of the tag relative to the robot instead of the environment. In this configuration, the positioning error can increase because the tag is located outside the convex hull made by the anchors [29]. The anchors installed in close proximity also negatively affect the positioning accuracy [30].

Assuming a configuration in which the human holds a UWB transceiver (tag) and four UWB transceivers are mounted on the mobile robot, this study proposes a novel method to estimate the position of the tag relative to the mobile robot for precise and robust human tracking in the following robot. Based on the previous LS approximation framework, the proposed method improves the positioning accuracy by developing sensor calibration to manage device-dependent error characteristics, as well as the interactive complementary filter to reduce the standard deviation of positioning errors. The main contribution of this study is the novel component-wise localization error correction method that alleviate not only the bias but also the deviation in the localization error. This paper identified two main components of localization error in the LS approximation through the mathematical derivation and proposed the error correction methods that alleviate the errors component by component. As the proposed method precisely tackles each error component based on in-depth error analysis, it achieved superior localization performance compared to the methods proposed in the literature. The contributions of this paper are as follows:

1. This study proposes a component-wise error correction method to increase the accuracy of UWB localization based on the in-depth analysis of UWB error characteristics. The core UWB error components are classified into two parts, i.e., bias and noise, and error corrections that fit into each component are proposed.
2. The sensor calibration is proposed to measure and compensate the device-dependent bias errors, which consistently appear in UWB localization. By conducting the calibration only once in the initialization, it can successfully correct the consistent bias of the localization.
3. The interactive complementary filter is proposed to alleviate the high-frequency noise of the localization. Radial and tangential components of the localization noise are significantly reduced by the proposed filter.

The following manuscript is structured as follows. In Section 2, the component-wise error correction method is described in detail including mathematical descriptions on the localization error analysis, initial calibration to correct bias error, and iterative complementary filter to alleviate deviation of the error. In Section 3, simulation and experimental results are presented to evaluate the performance of the proposed method. In Section 4, we discuss the advantages and limitations and conclude the paper in the following section.

## 2. Materials and Methods

### 2.1. Target Localization Using Least Square Approximation

**Figure 1:**
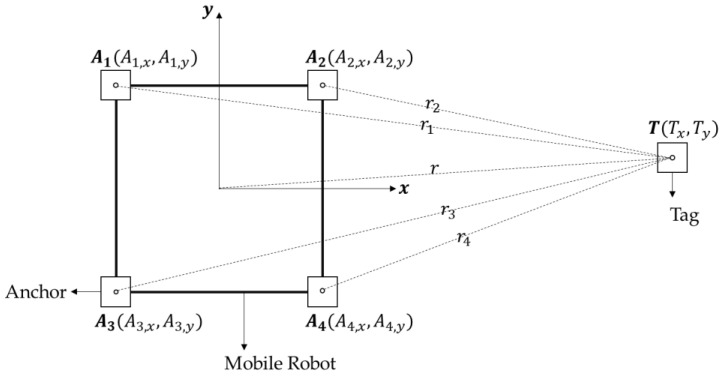
Overall configuration of UWB transceivers for the target localization.

Figure 1 shows the overall configuration of the UWB transceiver modules for target localization that should be followed by the robot. The position of the target is obtained relative to the robot-attached coordinate system. Four UWB transceiver modules, which are called anchors, are installed on the upper corners of the robot. The anchors are rigidly attached on the robot; thus, the coordinate values of the anchors with respect to the robot-attached coordinate system can be assumed to be constant. The other transceiver module, which is called the tag, is assumed to be held by the target, and the target position is estimated as the position of the tag. Four distance values, between the tag and each anchor, are measured using two-way ranging, and these values are given as inputs to the localization algorithm [31]. The distances can be expressed using the position of the tag and anchors as follows:

(1) $$r_{i}={\{\left(T-A_{i}\right){\left(T-A_{i}\right)}^{T}\}}^{\frac{1}{2}},i=1,2,3,4$$

where $r_{i}$ denotes the distance between the tag and the *i*th anchor, and $T={\left[T_{x}\;,\;T_{y}\right]}^{T}$, $A_{i}={\left[A_{i,x}\;,\;A_{i,y}\right]}^{T}$ are the position of the tag and *i*th anchor, respectively. By substituting $r^{2}=T_{x}^{2}+T_{y}^{2}$ into Equation (1), the following is obtained:

(2) $$T_{x}A_{i,x}+T_{y}A_{i,y}-\frac{1}{2}r^{2}=\frac{1}{2}\left(A_{i,x}{}^{2}+A_{i,y}{}^{2}-r_{i}{}^{2}\right)$$

Equation (2) can be expressed in a matrix form as

(3) AX=C

where

A=[A1,xA1,y−12⋮⋮⋮A4,xA4,y−12],X=[TxTyr2],C=12[A1,x2+A1,y2−r12⋮A4,x2+A4,y2−r42].

Because there exist measurement errors in $r_{i}$, X can be estimated using the LS approximation [32]:

(4) $$\overset{˜}{X}=arg\underset{\overset{¯}{X}}{\min}{\left(A\overset{¯}{X}-\overset{˜}{C}\right)}^{T}\left(A\overset{¯}{X}-\overset{˜}{C}\right)={\left(A^{T}A\right)}^{-1}A^{T}\overset{˜}{C},$$

where $\overset{˜}{X}=\left[{\tilde{T}}_{x},{\tilde{T}}_{y},{\tilde{r}}^{2}\right]$ is the estimated vector that represents the tag position and distance from the robot, and $\overset{¯}{X}=\left[{\overset{¯}{T}}_{x},{\overset{¯}{T}}_{y},{\bar{r}}^{2}\right]$ represents an optimization variable vector. In the vector $\overset{˜}{C}$, $r_{i}$ is replaced with ${\tilde{r}}_{i}$, which includes the measurement error.

### 2.2. Localization Error Analysis

Assuming that the coordinate system is located at the center of the robot, the coordinate of the anchor position $\left(A_{i,x},\;A_{i,y}\right)$ can be replaced with the width $\left(w_{x},\;w_{y}\right)$ of the robot, and Equation (4) can be expressed as

(5) $$\overset{˜}{X}=\left[\begin{array}{c} {\tilde{T}}_{x}\\ {\tilde{T}}_{x}\\ {\tilde{r}}^{2} \end{array}\right]={\left(A^{T}A\right)}^{-1}A^{T}\overset{˜}{C}=\left[\begin{array}{c} \frac{{\tilde{r}}_{1}^{2}-{\tilde{r}}_{2}^{2}-{\tilde{r}}_{3}^{2}+{\tilde{r}}_{4}^{2}}{4w_{x}}\\ \frac{{\tilde{r}}_{1}^{2}+{\tilde{r}}_{2}^{2}-{\tilde{r}}_{3}^{2}-{\tilde{r}}_{4}^{2}}{4w_{y}}\\ \frac{{\tilde{r}}_{1}^{2}+{\tilde{r}}_{2}^{2}+{\tilde{r}}_{3}^{2}+{\tilde{r}}_{4}^{2}-w_{x}^{2}-w_{y}^{2}}{4} \end{array}\right]\;$$

where ${\tilde{r}}_{i}=r_{i}+e_{i}$ represents the measured distance including the measurement error $e_{i}$. The estimation error of the tag position $E=\overset{˜}{T}-T$ can be expressed by substituting Equation (3) and ${\tilde{r}}_{i}=r_{i}+e_{i}$ into Equation (5):

(6) $$E=\left[\begin{array}{c} \frac{e_{1}^{2}-e_{1}^{2}-e_{1}^{2}+e_{1}^{2}+2\left(e_{1}r_{1}-e_{2}r_{2}-e_{3}r_{3}+e_{4}r_{4}\right)}{4w_{x}}\\ \frac{e_{1}^{2}+e_{1}^{2}-e_{1}^{2}-e_{1}^{2}+2\left(e_{1}r_{1}+e_{2}r_{2}-e_{3}r_{3}-e_{4}r_{4}\right)}{4w_{y}} \end{array}\right],\;$$

where $e_{i}$ denotes the measurement error between the tag and the *i*th anchor. Equation (6) indicates that the absolute value of the estimation error increases proportionally to the distance between the tag and the anchors ($r_{i}$), and it is inversely proportional to the distances between the anchors ($w_{x}$ and $w_{y}$). Therefore, the estimation error in the proposed configuration, in which the anchor placement is quite limited by the size of the robot, may be more amplified compared with the conventional configuration. Assuming that the square of the measurement errors ($e_{i}^{2})$ are negligible, and the size of the robot ($w_{x}$, $w_{y}$) is sufficiently small compared with the distance between the tag and the robot ($r_{i}\approx r)$, the estimation error can be simplified as

(7) $$E\approx \overset{˜}{E}=\left[\begin{array}{c} \frac{\left(e_{1}-e_{2}-e_{3}+e_{4}\right)}{2w_{x}}\\ \frac{\left(e_{1}+e_{2}-e_{3}-e_{4}\right)}{2w_{y}} \end{array}\right]r.\;$$

**Figure 2:**
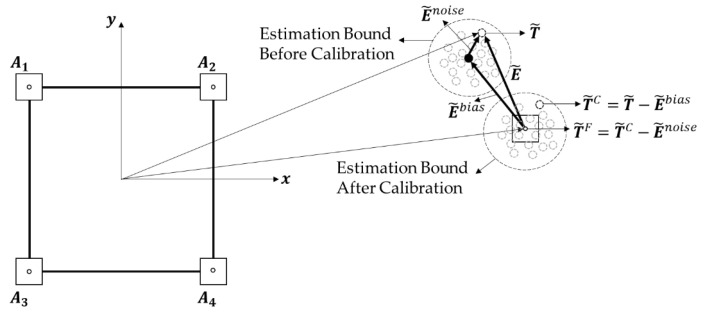
Component-wise classification of the estimation error. $\overset{˜}{T}$ denotes the initial estimation using the LS approximation of the tag position. ${\overset{˜}{T}}^{C}$ denotes the calibrated estimation by removing the bias error ${\overset{˜}{E}}^{bias}$ through initial calibration, and ${\overset{˜}{T}}^{F}$ denotes the final estimation, which removes ${\overset{˜}{E}}^{noise}$ from ${\overset{˜}{T}}^{C}$.

Assuming a normal distribution of $e_{i}$, the measurement error can be divided into two components as $e_{i}=e_{i}^{bias}+e_{i}^{noise}$, where $e_{i}^{bias}$ is the bias of the measurement, which can be regarded as the average of the measurement errors, and $e_{i}^{noise}$ represents the variation of the errors based on the central limit theorem [33]. Substituting this into Equation (7), the estimation error can be divided into two components as follows:

(8) $$\overset{˜}{E}=\left[\begin{array}{c} \frac{\left(e_{1}^{bias}-e_{2}^{bias}-e_{3}^{bias}+e_{4}^{bias}\right)}{2w_{x}}\\ \frac{\left(e_{1}^{bias}+e_{2}^{bias}-e_{3}^{bias}-e_{4}^{bias}\right)}{2w_{y}} \end{array}\right]r+\left[\begin{array}{c} \frac{\left(e_{1}^{noise}-e_{2}^{noise}-e_{3}^{noise}+e_{4}^{noise}\right)}{2w_{x}}\\ \frac{\left(e_{1}^{noise}+e_{2}^{noise}-e_{3}^{noise}-e_{4}^{noise}\right)r}{2w_{y}} \end{array}\right]r={\overset{˜}{E}}^{bias}+{\overset{˜}{E}}^{noise},\;$$

where ${\overset{˜}{E}}^{bias},\;{\overset{˜}{E}}^{noise}$ are fractions of the estimation errors that represent the bias and noise components, respectively. Figure 2 shows the component-wise classification of the estimation error.

### 2.3. Component-Wise Estimation Error Correction

This study proposes a component-wise error correction strategy that mitigates two components of the estimation error, i.e., ${\overset{˜}{E}}^{bias}$, ${\overset{˜}{E}}^{noise}$, to improve the accuracy of the target localization. The final estimation of the tag position ${\overset{˜}{T}}^{F}$ is computed by removing the estimation error component by component as follows:

(9) $${\overset{˜}{T}}^{F}={\overset{˜}{T}}^{C}-{\overset{˜}{E}}^{noise}=\overset{˜}{T}-{\overset{˜}{E}}^{bias}-{\overset{˜}{E}}^{noise},$$

where $\overset{˜}{T}$ is the initial estimation of the tag position using the LS approximation without error correction, and ${\overset{˜}{T}}^{C}$ is the calibrated estimation that removes ${\overset{˜}{E}}^{bias}$ from $\overset{˜}{T}$. ${\overset{˜}{T}}^{F}$ is the final estimation that removes both error components, ${\overset{˜}{E}}^{bias}$ and ${\overset{˜}{E}}^{noise}$.

#### 2.3.1. Bias Correction through Initial Calibration

**Figure 3:**
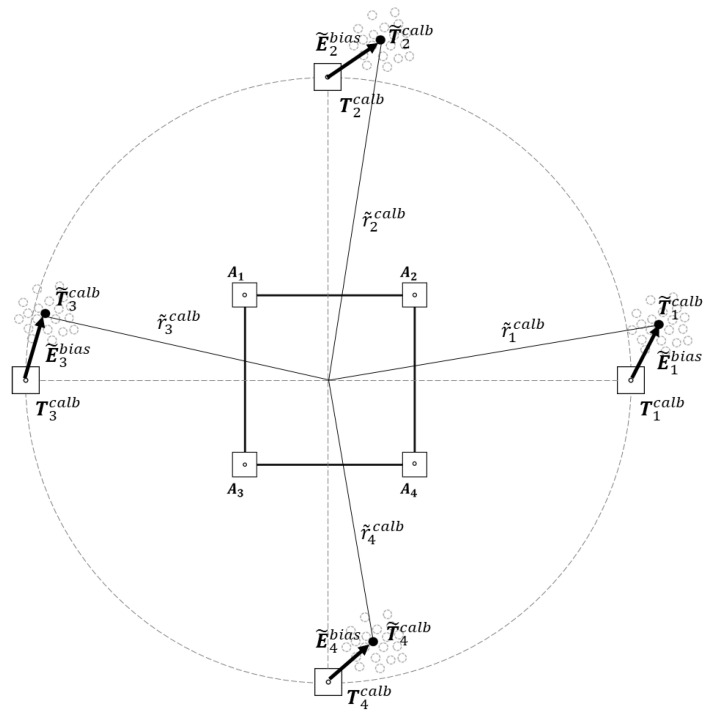
Initial calibration method to measure bias error ${\overset{˜}{E}}^{bias}$. Initial calibration is conducted prior to the measurement at multiple calibration points to measure ${\overset{˜}{E}}_{norm}^{bias}$.

The bias error ${\overset{˜}{E}}^{bias}$ arises from the bias of the distance measurements $e_{i}^{bias}$, as shown in Equation (8), and it can be regarded as the device-dependent error component. Assuming that ${\overset{˜}{E}}^{bias}$ is a static offset that varies according to the UWB transceiver modules (specific pair of tag and anchor), ${\overset{˜}{E}}^{bias}$ can be corrected through the initial calibration, which needs to be conducted once prior to the measurement. In the initial calibration, samples of the tag positions are collected using a standard LS approximation at pre-determined positions for a particular period. The bias error is computed as ${\overset{˜}{E}}^{bias}=T^{calb}-{\overset{˜}{T}}_{ave}^{calb}$, where $T^{calb}$ denotes the true position of the calibration point, and ${\overset{˜}{T}}_{ave}^{calb}$ represents the average of the estimation samples acquired at the calibration point. Because ${\overset{˜}{E}}^{bias}$ varies according to the distance r, a normalized bias error ${\overset{˜}{E}}_{norm}^{bias}$= ${\overset{˜}{E}}^{bias}/r$ is used in the calibration. The calibration should be performed at multiple calibration points, as shown in Figure 3, to reflect the error characteristics of the UWB transceiver, which vary according to the direction of the signal [34]. ${\overset{˜}{E}}_{norm}^{bias}$ is computed as the average of the normalized biases measured at the calibration points as follows:

(10) $${\overset{˜}{E}}_{norm}^{bias}=\frac{1}{n}\sum_{i=1}^{n}\left(\frac{T_{i}^{calb}-{\overset{˜}{T}}_{i,\;ave}^{calb}}{{\tilde{r}}_{i}^{calb}}\right),\;$$

where $T_{i}^{calb}$, ${\overset{˜}{T}}_{i,\;ave}^{calb}$ represent the true and estimated position of the *i*th calibration point, ${\tilde{r}}_{i}^{calb}$ denotes the distance between the robot and ${\overset{˜}{T}}_{i,\;ave}^{calb}$, and n is the number of calibration points. Then, the calibrated tag position ${\overset{˜}{T}}^{C}$ can be computed from the initial estimation $\overset{˜}{T}$ as follows:

(11) $${\overset{˜}{T}}^{C}=\overset{˜}{T}-{\overset{˜}{E}}^{bias}=\overset{˜}{T}-{\overset{˜}{E}}_{norm}^{bias}\times \tilde{r},\;$$

where $\tilde{r}$ denotes the estimated distance between the robot and tag computed by the LS approximation.

#### 2.3.2. Noise Reduction Using Iterative Complementary Filter

**Figure 4:**
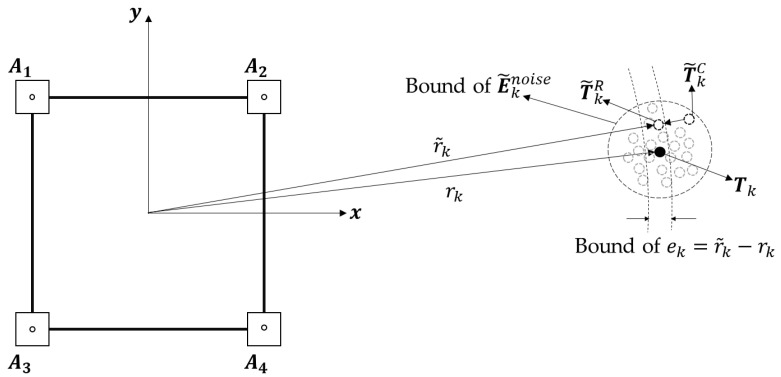
Alleviating the radial directional noise component of ${\overset{˜}{E}}_{k}^{noise}$. The magnitude of ${\overset{˜}{T}}_{k}^{C}$ is adjusted by the distance estimation ${\tilde{r}}_{k}$.

${\overset{˜}{E}}^{noise}$ arises from the deviation in the distance measurement ($e_{i}^{noise}$), and it has high-frequency characteristics that can be alleviated by low-pass filters. This study proposes an iterative complementary filter (ICF), which acts similar to a low-pass filter to remove high-frequency noise components in $\overset{˜}{T}$. The main idea of the ICF is combining the previous and current estimation with variable weight coefficients, which are determined by evaluating the reliability of the estimation. The noise-filtering procedure of the ICF is composed of the following three steps:

STEP 1 (*Alleviating Radial Directional Noise Components*): The calibrated estimation at time *k* (${\overset{˜}{T}}_{k}^{C}$) can be further improved by adjusting its magnitude using the distance estimation ${\tilde{r}}_{k}$**,** which is acquired by the LS approximation. The subscript *k* indicates the sampling time of the variables. While ${\overset{˜}{E}}_{k}^{noise}$ is amplified by the distance $r_{k}$ (Equation (8)), the errors in the distance estimation ${\tilde{r}}_{k}$ are determined only by linear combination of the distance errors between the tag and anchors $e_{i,k}$. The magnitude and variation of the distance error are smaller than those of ${\overset{˜}{E}}_{k}^{noise}$, and it can be used to alleviate the radial direction component of ${\overset{˜}{E}}_{k}^{noise}$ included in ${\overset{˜}{T}}_{k}^{C}$, as shown in Figure 4. The estimation corrected in the radial direction is computed as follows:

(12) $${\overset{˜}{T}}_{k}^{R}={\tilde{r}}_{k}\times \frac{{\overset{˜}{T}}_{k}^{C}}{{\overset{˜}{T}}_{k}^{C}}\;$$

where ${\overset{˜}{T}}_{k}^{R}$ denotes the estimation corrected in the radial direction at time step *k*.

**Figure 5:**
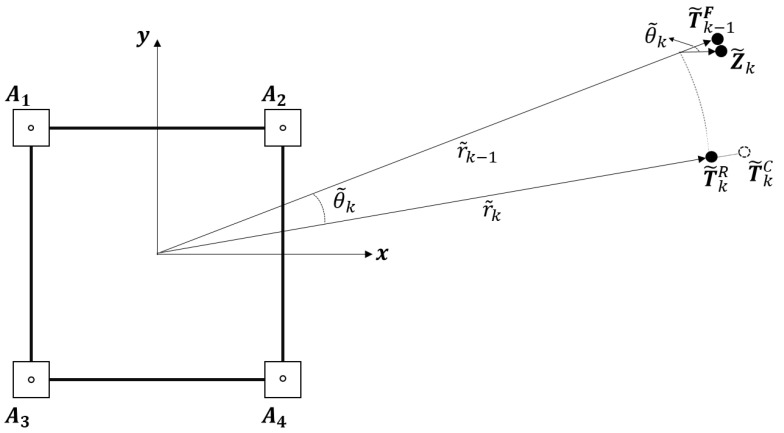
Computing estimation candidate ${\tilde{Z}}_{k}$ from previous estimation ${\overset{˜}{T}}_{k-1}^{F}$.

STEP 2 (*Computing Estimation Candidate*${\tilde{Z}}_{k}$*from Previous Estimation*${\overset{˜}{T}}_{k-1}^{F}$): ${\overset{˜}{T}}_{k}^{R}$ computed in *STEP1* is one part of the final estimation ${\overset{˜}{T}}_{k}^{F}$. The other part ${\tilde{Z}}_{k}$ is computed by attracting the previous estimation ${\overset{˜}{T}}_{k-1}^{F}$ to ${\overset{˜}{T}}_{k}^{R}$, as shown in Figure 5. The estimation candidate ${\tilde{Z}}_{k}$ is computed as follows:

(13) $${\tilde{Z}}_{k}={\tilde{r}}_{k}\frac{{\overset{˜}{T}}_{k-1}^{F}}{r_{k-1}^{F}}+\left({\tilde{r}}_{k-1}^{F}-{\tilde{r}}_{k}\right)\frac{{\overset{˜}{T}}_{k}^{R}}{{\tilde{r}}_{k}}{\tilde{r}}_{k},\;$$

where $r_{k-1}^{F}=\|{\overset{˜}{T}}_{k-1}^{F}\|$ denotes the distance between the robot and the previous estimation of the tag position.

STEP 3 (*Computing Final Estimation*${\overset{˜}{T}}_{k}^{F}$): The final estimation of the tag position ${\overset{˜}{T}}_{k}^{F}$ is computed as a weighted linear combination of ${\overset{˜}{T}}_{k}^{R}$ and ${\tilde{Z}}_{k}$ as follows:

(14) $${\overset{˜}{T}}_{k}^{F}=\frac{\alpha_{k}{\tilde{Z}}_{k}+\beta_{k}{\overset{˜}{T}}_{k}^{R}}{\alpha_{k}+\beta_{k}}\;,$$

where $\alpha_{k},\;\beta_{k}$ are the weight coefficients of the ICF that represent the reliability of ${\tilde{Z}}_{k}$ and ${\overset{˜}{T}}_{k}^{R}$, respectively, and can be determined as follows:

(15) $$\alpha_{k}=\frac{1}{4}\sum_{i=1}^{4}\left|{\left\{\left({\overset{˜}{T}}_{k}^{R}-A_{i}\right){\left({\overset{˜}{T}}_{k}^{R}-A_{i}\right)}^{T}\right\}}^{\frac{1}{2}}-{\tilde{r}}_{i}\right|,\;\beta_{k}=\frac{1}{4}\sum_{i=1}^{4}\left[{\left\{\left({\tilde{Z}}_{k}-A_{i}\right){\left({\tilde{Z}}_{k}-A_{i}\right)}^{T}\right\}}^{\frac{1}{2}}-{\tilde{r}}_{i}\right]\;$$

The reliability of the ${\tilde{Z}}_{k}$ and ${\overset{˜}{T}}_{k}^{R}$ values is evaluated by comparing the estimated and measured distance between the anchor and tag.

## 3. Results

**Figure 6:**
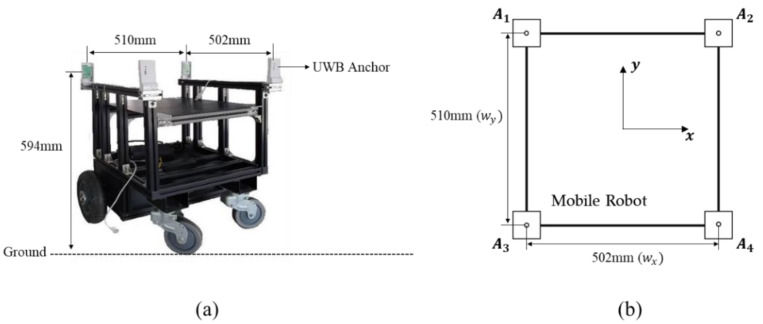
Configuration of UWB anchors used in simulations and experiments for localization-accuracy evaluation: (**a**) UWB anchors attached to the mobile robot, (**b**) UWB anchor configurations with attached coordinate system and dimensions.

The localization accuracy of the proposed method was validated via simulation and experiment. Figure 6 shows the configuration of the UWB anchors attached to the mobile robot (OMO-R1, OMOROBOT Inc., Gyeonggi-do, Korea) that was used for the simulation and experiment. As shown in the figure, a UWB anchor is mounted on each of the four corners of the robot. The UWB anchors are 594 mm above ground, and the distance between them is set to 502 ($w_{x}$) and 510 mm ($w_{y}$). Using these UWB anchors, we evaluated the localization accuracy of the proposed method at various tag positions.

### 3.1. Simulation Setup and Results

#### 3.1.1. Simulation Setup

The simulation was constructed using MATLAB R2021 (MathWorks, Natick, MA, USA). The distance-measurement error between the UWB anchor and tag is defined by the mean and standard deviation. These values were acquired through the experiment, in which the distance-measurement data was collected for a minute at four reference points (0°, 90°, 180°, 270°) located 1 m away from the center of the robot. The mean and standard deviation of the error used in the simulation are shown in Table 1.

**Figure 7:**
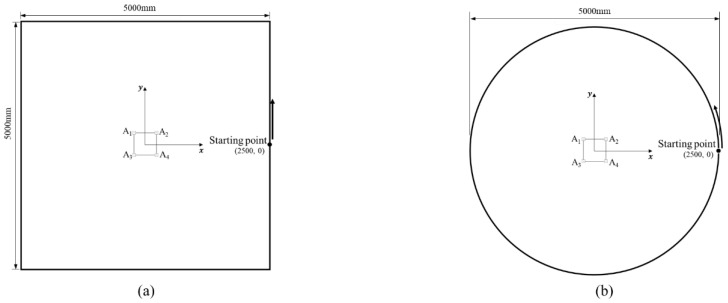
Two reference paths used in the simulation: (**a**) square path with a side length of 5 m, (**b**) circular path with a diameter of 5 m.

Figure 7 shows two simulation scenarios used for the accuracy evaluation. Assuming that the robot is fixed, the tag moves following two different reference paths around the robot. A square path with a side length of 5000 mm and a circular path with a diameter of 5000 mm were used as reference paths. In both scenarios, the robot was located at the center of the paths, and the tag passed through the entire path one time from the starting point (2500, 0) with constant velocity of 100 mm/s. The height of the tag was set as 594 mm from the ground which is identical to the height of the anchor. A total of 2000 samples of the tag-position estimation were acquired for each path.

#### 3.1.2. Simulation Results

**Figure 8:**
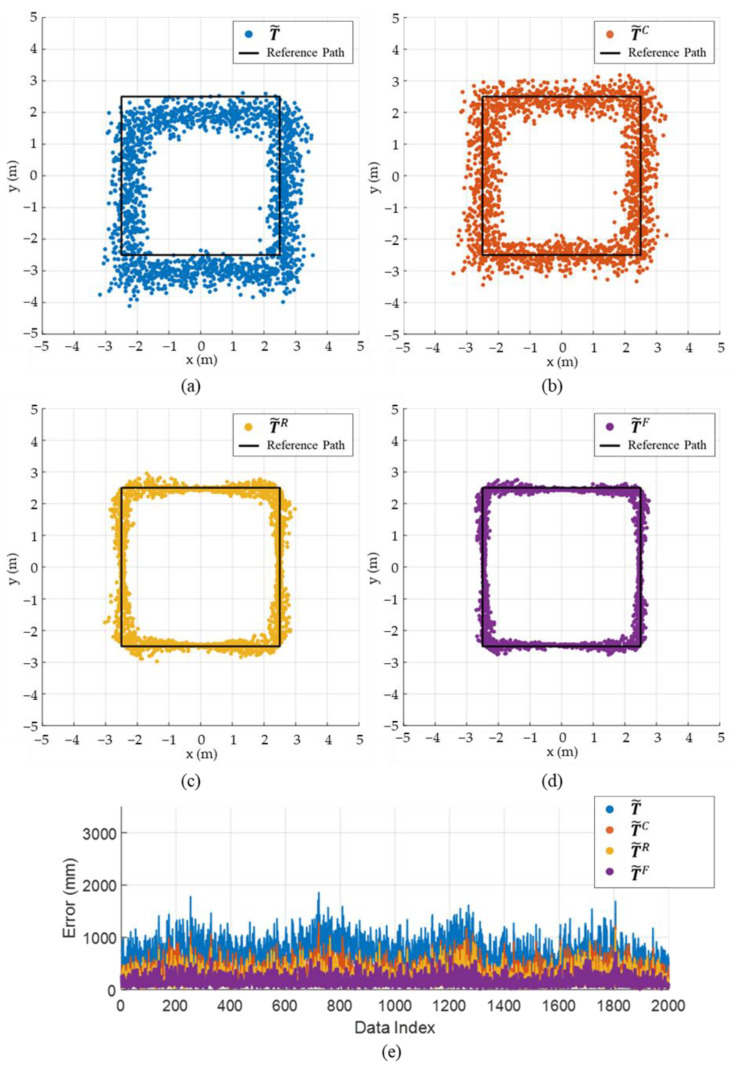
Simulation results demonstrating the estimation accuracy of the proposed method for the square path: (**a**) estimation results of $\overset{˜}{T}$**,** (**b**) estimation results of ${\overset{˜}{T}}^{C}$, (**c**) estimation results of ${\overset{˜}{T}}^{R}$, (**d**) estimation results of ${\overset{˜}{T}}^{F}$, (**e**) comparison of estimation error for $\overset{˜}{T},\;{\overset{˜}{T}}^{C},\;{\overset{˜}{T}}^{R},\;and\;{\overset{˜}{T}}^{F}$.

**Figure 9:**
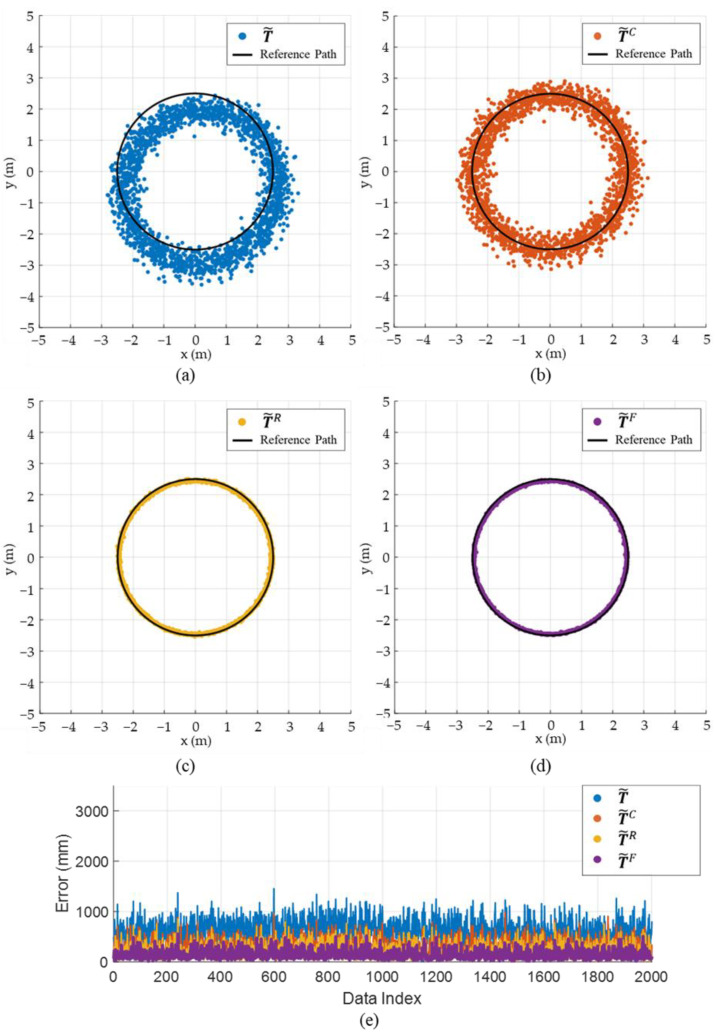
Simulation results demonstrating the estimation accuracy of the proposed method for the circular path: (**a**) estimation results of $\overset{˜}{T}$**,** (**b**) estimation results of ${\overset{˜}{T}}^{C}$, (**c**) estimation results of ${\overset{˜}{T}}^{R}$, (**d**) estimation results of ${\overset{˜}{T}}^{F}$, (**e**) comparison of estimation error for $\overset{˜}{T},\;{\overset{˜}{T}}^{C},\;{\overset{˜}{T}}^{R},\;\mathrm{and}\;{\overset{˜}{T}}^{F}$.

Figure 8 and Figure 9 shows the effect of component-wise error removal for the square and circular paths, respectively. The result of the position estimation using the LS approximation ($\overset{˜}{T}$) (Figure 8a and Figure 9a) shows a consistent bias to the right-lower direction for both paths. The initial bias was successfully calibrated by applying the initial calibration (${\overset{˜}{T}}^{C}$) as it removes the ${\overset{˜}{E}}^{bias}$ component, as shown in Figure 8b and Figure 9b. The variation of the error was alleviated by removing the radial-direction noise component (${\overset{˜}{T}}^{R}$), as shown in Figure 8c and Figure 9c, and the errors were further reduced by applying the ICF (${\overset{˜}{T}}^{F}$), as shown in Figure 8d and Figure 9d. Figure 8e and Figure 9e confirm that the estimation errors were improved stepwise as the errors were alleviated component by component. Table 2 shows the mean, standard deviation, maximum, and minimum values of the estimation error of $\overset{˜}{T},\;{\overset{˜}{T}}^{C},\;{\overset{˜}{T}}^{R},\;and\;{\overset{˜}{T}}^{F}$ in the square path. The mean errors were 703.3, 360.2, 239.5, and 162.2 mm for $\overset{˜}{T},\;{\overset{˜}{T}}^{C},\;{\overset{˜}{T}}^{R},\;and\;{\overset{˜}{T}}^{F}$, respectively. The proposed method improves the mean and standard deviation of the estimation error of the LS approximation by up to 77 and 62%, respectively, for the square path. Table 3 shows the estimation error for the circular path, which is similar to the results of the square path.

**Figure 10:**
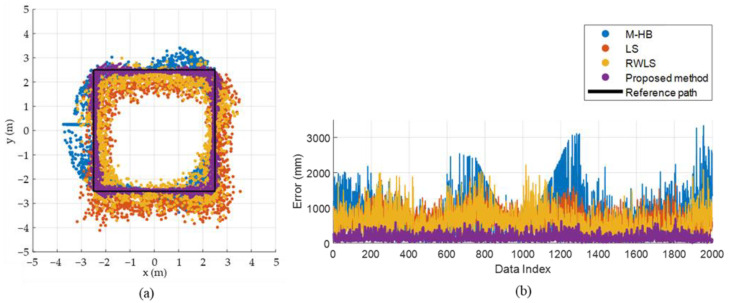
Comparison of estimation accuracy between the proposed method and previous methods for the square path: (**a**) comparison of estimation results, (**b**) comparison of estimation error.

**Figure 11:**
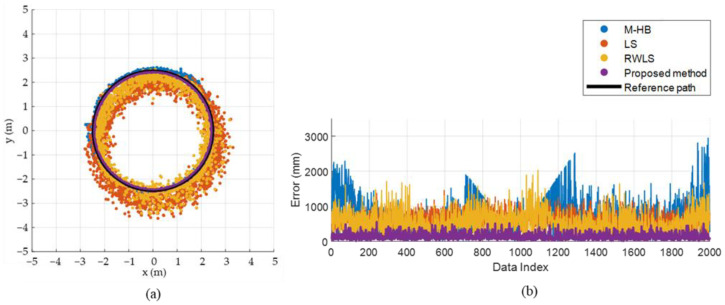
Comparison of estimation accuracy between the proposed method and previous methods for the circular path: (**a**) comparison of estimation results, (**b**) comparison of estimation error.

Figure 10 and Figure 11 show the estimation accuracy of the proposed method compared with previous methods, i.e., LS [32], RWLS [27], and M-HB [28], in the square and circular paths, respectively. The proposed method exhibits a superior performance in bias correction and alleviates the variation because the estimation result of the proposed method is located in the closest region to the reference path compared with the previous methods. The proposed method improved the mean estimation error by up to 79.8, 76.9, and 74.0% compared with the M-HB, LS, and RWLS methods, respectively, for the square path, as shown in Table 4. Similarly, the standard deviation also improved by up to 81.7, 61.9, and 70.5% compared with the M-HB, LS, and RWLS methods, respectively. For the circular path, the results were similar to those of the square path, as presented in Table 5.

### 3.2. Experimental Setup and Results

#### 3.2.1. Experimental Setup

**Figure 12:**
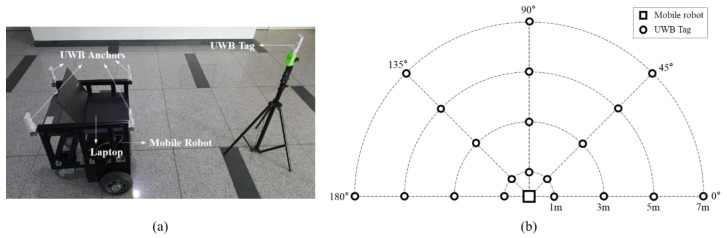
Experimental setup for performance evaluation: (**a**) experimental setup, (**b**) reference positions of the UWB tag.

Figure 12 shows an experimental setup to evaluate the estimation accuracy of the proposed method. The experiment was conducted using the UWB transceiver module DWM1001 (Decawave, Ireland). It uses 38.4 MHz reference crystal and has about 20 cm ranging error [35,36]. The UWB channel and data rate were set as 2 (3.99 GHz) and 6.8 Mbps, respectively. The UWB anchor configuration was identical to the simulation setup (Figure 6). The height of the tag was set as 1000 mm from the ground considering the height of the tag held by the person in general situation. Since the heights of the anchors and the tag were different, the distance measurement between the tag and each anchor were adjusted by projecting it on the plane made by the anchors. The estimation accuracy was evaluated at 20 pre-defined reference locations with various distances (1, 3, 5, 7 m) and angles (0°, 45°, 90°, 135°, 180°) from the robot, as shown in Figure 12b. Four distances, measured for each pair of UWB anchor and tag, were collected at the master anchor (one of four anchors) and transmitted to a laptop through serial communication. The communication rate between the master anchor and laptop was 6–7 Hz. The localization algorithms, including the proposed and previous methods, were executed on the laptop to compute the position estimation of the tag using the four given distance values. Approximately 400 sets of samples were collected at each reference location and used to compute the position of the tag. The initial calibration was performed prior to conduct the experiment with the UWB anchors and tag modules used in the experiment.

#### 3.2.2. Experimental Results

**Figure 13:**
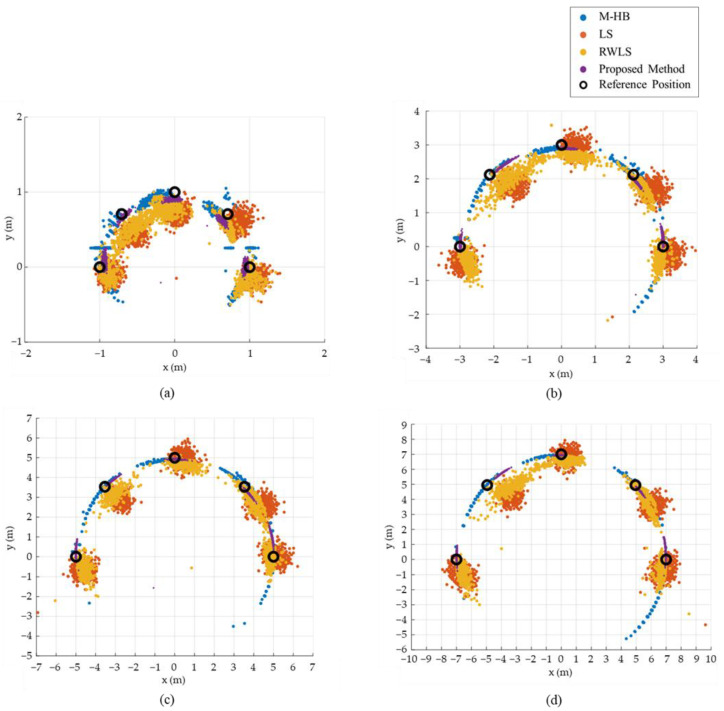
Experimental setup for performance evaluation. (**a**) experimental setup, (**b**–**d**) reference locations.

The performance of the proposed method for each step was analyzed through the simulation. Therefore, the final result of the proposed method (${\overset{˜}{T}}^{F}$) will only be discussed as a comparison with the previous methods. The position-estimation results for each method are plotted in Figure 13, and the mean, standard deviation, maximum, and minimum values at four different distances are listed in Table 6. The mean and standard deviation of the error were improved for all distances (1, 3, 5, 7 m) compared with the previous methods. The proposed method exhibited an improvement of 50.56% for the mean and 38.98% for the standard deviation compared with the LS approximation.

## 4. Discussion

In the proposed UWB localization method, the anchors are mounted in a narrow region constrained by the size of the mobile robot, and the tag is positioned outside of the convex hull of the anchors. This results in large errors that are proportional to the distance between the tag and the anchors. To overcome this disadvantageous UWB configuration and to enhance localization accuracy, this study proposed component-wise error corrections that effectively alleviate device-dependent bias and high-frequency noise component by component based on the in-depth analysis of localization errors. The experimental validation shows that the localization errors were considerably improved by up to 50.6% by applying the proposed initial calibration and the ICF compared with the conventional LS approximation.

The UWB localization errors originate from the errors in the distance measurement between the anchor and tag pair, which varies depending on both the device and antenna orientations. Hence, calibrating the distance-measurement errors for each anchor and tag pair cannot effectively remove the resultant localization bias. This study proposed an initial calibration method to measure the localization bias, which is specific to the given anchor configuration, not to a single anchor module. The bias is measured at four reference positions, in which the tag is located in four different directions from the anchors, to consider the variation in the bias errors depending on the antenna orientation. Furthermore, normalization of the bias enables the calibration of the bias error, not only in various directions, but also at various distances to the tag position. The bias corrections made by the proposed calibration method considerably improved the localization error of the conventional LS approximation by up to 49% in the simulation results.

Although the initial calibration effectively corrects bias errors, noise-like high-frequency error components still remain in the localization. The ICF is proposed to eliminate the high-frequency error component (${\overset{˜}{E}}^{noise}$). Assuming that the distance estimation of the LS approximation is more reliable than the localization, the bias-removed tag position is adjusted in the radial direction before applying the iterative filter. The radial directional deviations were significantly reduced in this step. The remaining error components were alleviated by iteratively updating the current estimation as a weighted sum of the previous and current estimation. In this iterative procedure, the weight values were flexibly determined by evaluating the reliability of each estimation. The ICF contributed to the improvement in the variation of the error, and the standard deviation of the error was substantially improved by up to 62% in the simulation results.

The proposed component-wise error correction method significantly improved target localization accuracy by up to 77 and 51% compared with the LS approximation in the simulation and experimental validation, respectively; however, there is still a limitation that should be addressed in future research. In this paper, the effect of the human, who hold the tag, or any other obstacles causing non-line-of-sight (NLOS) situations to the localization accuracy. We have conducted pilot experiment to identify the human effect to the localization accuracy. The experimental results reveal that the human effect was not significant, but it requires in-depth analysis and additional experiments to prove practical applicability of the proposed method. Although the magnitude and deviation of the error were reduced compared with previous methods, the error still increases as the tag position moves farther away from the anchor, as shown in Table 6. This error characteristic is inherent to the UWB configuration of the following robot, and it can be improved using additional sensors, such as an inertial measurement unit (IMU). We believe that sensor fusion with the IMU can improve the localization performance of the proposed method in future research.

## 5. Conclusions

In this study, we proposed a component-wise error-correction method to improve the localization accuracy of a target-following mobile robot. The localization bias and high-frequency deviation of the error were successfully alleviated through the proposed initial calibration and iterative complementary filter. The performance evaluation conducted via the simulation and experiment confirmed that the proposed method significantly improves the localization accuracy of previous methods. We believe that the proposed method can be used for the industrial application of following robots, and it contributes to easing the burden of the human operator.

## Figures and Tables

**Table 1:** Mean and standard deviation of distance measurement used in the simulation.

UWB Anchor ID	Mean (mm)	Standard Deviation
A_1_	−10	50
A_2_	−180	50
A_3_	56	50
A_4_	−29	50

**Table 2:** Comparison of estimation error for $\overset{˜}{T}$, ${\overset{˜}{T}}^{C}$, ${\overset{˜}{T}}^{R}$ and ${\overset{˜}{T}}^{F}$ in the square path.

	Mean (mm)	SD (mm)	Max (mm)	Min (mm)
$\overset{˜}{T}$	703.3	283.0	1856.3	32.1
${\overset{˜}{T}}^{C}$	360.2	197.5	1286.6	3.7
${\overset{˜}{T}}^{R}$	239.5	171.6	1135.9	4.8
${\overset{˜}{T}}^{F}$	162.2	107.8	694.8	4.5

**Table 3:** Comparison of estimation error for $\overset{˜}{T}$, ${\overset{˜}{T}}^{C}$, ${\overset{˜}{T}}^{R}$ and ${\overset{˜}{T}}^{F}$ in the circular path.

	Mean (mm)	SD (mm)	Max (mm)	Min (mm)
$\overset{˜}{T}$	610.2	235.9	1453.1	4.0
${\overset{˜}{T}}^{C}$	314.8	162.0	979.0	15.1
${\overset{˜}{T}}^{R}$	212.8	146.5	849.3	3.9
${\overset{˜}{T}}^{F}$	143.3	91.2	552.9	11.0

**Table 4:** Comparison of estimation error between the proposed method and previous methods for the square path.

	Mean (mm)	SD (mm)	Max (mm)	Min (mm)
M-HB	801.8	588.1	3334.1	9.4
LS	703.3	283.0	1856.3	32.1
RWLS	623.9	365.8	2212.7	7.0
Proposed Method	162.2	107.8	694.8	4.5

**Table 5:** Comparison of estimation error between the proposed method and previous methods for the circular path.

	Mean (mm)	SD (mm)	Max (mm)	Min (mm)
M-HB	686.1	495.5	2945.7	3.8
LS	610.2	235.9	1453.1	4.0
RWLS	547.6	312.7	2031.7	11.8
Proposed Method	143.3	91.2	552.9	11.0

**Table 6:** Comparison of estimation error of the proposed method with that of the previous methods for the circular path.

Distance	Method	Mean (mm)	SD (mm)	Max (mm)	Min (mm)
1 m	M-HB	274.8	117.2	754.4	33.8
	LS	242.7	76.1	612.6	58.1
	RWLS	219.6	88.7	547.4	25.8
	Proposed Method	96.6	36.2	429.4	36.4
3 m	M-HB	610.0	313.4	1546.8	111.1
	LS	592.3	189.7	1442.5	109.4
	RWLS	560.1	281.6	1884.2	63.6
	Proposed Method	282.3	112.4	844.8	64.6
5 m	M-HB	976.1	529.7	3851.6	61.0
	LS	863.3	341.0	4026.0	186.9
	RWLS	676.0	378.4	2825.7	36.1
	Proposed Method	487.9	216.3	2280.7	113.2
7 m	M-HB	1303.6	573.5	3737.7	138.6
	LS	1271.6	412.9	4083.6	185.4
	RWLS	1132.1	601.4	3413.9	100.0
	Proposed Method	601.3	257.3	2127.7	139.0

## Data Availability

The data presented in this study are available in insert article.
